# Note Taking

**Published:** 1866-02

**Authors:** 


### NOTE TAKING.

Sir Benjamin Brodie says it was from his friend Jeffreys that he first learned the importance of keeping written notes of cases. All his life he kept notes. At the bedside of patients he jotted down a few memoranda, which he afterward expanded in the evening; and his notes of cases thus kept for half a century, now form many quarto volumes. He did not find that the habit of committing his observations to writing weakened his memory, but rather that it strengthened it; and in the most strenuous terms he insists that no one can become thoroughly well acquainted with his profession, either as a physician or as a surgeon, who has not studied it in that manner. He has always tried to impress this fact on his pupils, and has often lamented that only a small portion of them would follow his advice.—*Brit. Med. Journal.*

Dr. Richardson, an English chemist, says that iodine placed in a small box, with a perforated lid, destroys organic poison in rooms. During the continuance of an epedemic small-pox in London, he saw the method used with benefit.—*Amer. Druggists' Circular and Chemical Gazette.*

